# Evaluation of Left Ventricular Function Using Four-Dimensional Flow Cardiovascular Magnetic Resonance: A Systematic Review

**DOI:** 10.3390/jcdd9090304

**Published:** 2022-09-12

**Authors:** Jiaxing Jason Qin, Ben Indja, Alireza Gholipour, Mustafa Gök, Stuart M. Grieve

**Affiliations:** 1 Imaging and Phenotyping Laboratory, Charles Perkins Centre, University of Sydney, Sydney, NSW 2006, Australia; 2 Faculty of Medicine and Health, University of Sydney, Sydney, NSW 2006, Australia; 3 Department of Radiology, Faculty of Medicine, Aydin Adnan Menderes University, Aydin 09010, Turkey; 4 Department of Radiology, Royal Prince Alfred Hospital, Sydney, NSW 2006, Australia

**Keywords:** 4D-flow MRI, left ventricular function, flow quantification, clinical application, cardiovascular magnetic resonance

## Abstract

There is increasing recognition of the value of four-dimensional flow cardiovascular magnetic resonance (4D-flow MRI) as a potential means to detect and measure abnormal flow behaviour that occurs during early left ventricular (LV) dysfunction. We performed a systematic review of current literature on the role of 4D-flow MRI-derived flow parameters in quantification of LV function with a focus on potential clinical applicability. A comprehensive literature search was performed in March 2022 on available databases. A total of 1186 articles were identified, and 30 articles were included in the final analysis. All the included studies were ranked as “highly clinically applicable”. There was considerable variability in the reporting of methodologies and analyses. All the studies were small-scale feasibility or pilot studies investigating a diverse range of flow parameters. The most common primary topics of investigation were energy-related flow parameters, flow components and vortex analysis which demonstrated potentials for quantifying early diastolic dysfunction, whilst other parameters including haemodynamic forces, residence time distribution and turbulent kinetic energy remain in need of further evaluation. Systematic quantitative comparison of study findings was not possible due to this heterogeneity, therefore limiting the collective power of the studies in evaluating clinical applicability of the flow parameters. To achieve broader clinical application of 4D-flow MRI, larger scale investigations are required, together with standardisation of methodologies and analytical approach.

## 1. Introduction

Assessment of left ventricular (LV) function is a key component of multiple disease processes. LV functional assessment conventionally includes ventricular size and volumes, wall thickness, contractile function motion and strain. Impairment of LV function is an important prognostic marker and measure of treatment effect in valvular heart diseases, ischaemic heart disease, and other acquired or congenital causes of cardiomyopathy [1]. It is known that LV dysfunction is associated with significant changes in diastolic and systolic flow; hence, direct measurement of LV flow represents a good candidate for sensitive early detection of LV dysfunction.

Conventional quantification of LV function includes measurements of LV ejection fraction (LVEF); LV size metrics such as end diastolic volume (EDV), end systolic volume (ESV), stroke volume (SV); and myocardial parameters such as wall thickness, strain, or regional wall motion abnormalities. Transthoracic echocardiogram (TTE) is one of the most commonly used imaging modalities in clinical practice. However, TTE has inherent limitations in imaging quality and reproducibility due to patient factors and operator dependence. In addition, TTE is a two-dimensional (2D) modality which can limit its assessment of complex three-dimensional features within the LV [2]. The presence of valvular pathology may also influence the accuracy of LV function measurements [3]. Three-dimensional (3D) TTE has been shown to be superior to its 2D counterpart in quantifying LV volumes and LVEF; however, the technique remained limited in temporal and spatial resolutions and underestimated cardiac volumes compared to gold standard cardiac magnetic resonance (CMR) [4]. Transoesophageal echocardiogram (TOE) is able to obtain better image quality; however, it is an semi-invasive procedure requiring a team of medical specialists to perform and is therefore less readily available in routine clinical use [5]. Computed tomography (CT) is capable of acquiring high-resolution anatomical images, but is limited in functional evaluation and uses ionising radiation [6].

Increasingly, the potential of four-dimensional flow cardiovascular magnetic resonance (4D-flow MRI) in cardiovascular applications is being recognised [2,7]. Compared to conventional CMR, 4D-flow MRI has the added benefit of being a time-resolved, three-dimensional (3D) technique, and is capable of acquiring functional flow data in true 3D fashion in addition to anatomical data. Furthermore, 4D-flow MRI enables the visualisation and measurement of velocity vectors and flow paths, quantification of flow volumes as well as conventional and novel flow parameters [2]. In early LV dysfunction, remodelling may be absent or subtle and therefore not readily detected by conventional CMR measurements of LV volumes and EF, yet alterations in flow dynamics may be more evident [8]. As a result, 4D-flow measured flow parameters may be superior in detecting subtle early LV pathologies compared to conventional CMR, with potential benefits of early treatment and intervention. In addition, the quantification of flow as well as anatomical parameters will provide insights into the correlation between structure and function, adding to a more detailed understanding of cardiovascular function and pathologies [2,9]. However, current clinical utilisation of 4D-flow MRI remains limited, especially in the quantification of LV function. Several factors are important for clinical translation of new diagnostic techniques, including cost effectiveness, reproducibility, and diagnostic equivalence [10].

The aim of this systematic review is to examine the role of 4D-flow MRI to date in quantifying LV function using novel flow parameters and the challenges limiting its broader clinical applications. Recognising the heterogeneity within this field, we also aim to examine the acquisition, analysis and reporting differences in the available literature to evaluate inter-study comparability.

## 2. Materials and Methods

### 2.1. Systematic Review Registration

This systematic review was prospectively registered (CRD42022329941) with the international database of prospectively registered systematic reviews (PROSPERO).

### 2.2. Search Strategy

A systematic review of the literature was performed independently by JQ and BI with searches carried out in electronic databases (PubMed, Medline and Google Scholar) for relevant articles using the Preferred Reporting Items for Systematic Reviews and Meta-Analysis (PRISMA) checklist [11]. Key search terms included “Magnetic resonance imaging”, “ventricular function, Left”, “Heart ventricle/diagnostic imaging”, “Ventricular function/physiology”, “Ventricular dysfunction, Left”, “Magnetic resonance imaging, cine/methods”. Reference lists of relevant studies were also reviewed for further articles.

### 2.3. Eligibility Criteria

Included studies were those that used 4D-flow MRI to derive novel flow parameters to assess LV function in adult populations in healthy and diseased states. Exclusion criteria included studies with a focus on aortic, valvular, atrial or right ventricular (RV) blood flow patterns, studies that only investigated non-flow parameters such as LV mass, LV volumes, LVEF or conventional flow parameters such as velocity or flow volumes, studies with exclusively paediatric populations (<16 years), phantom studies, review articles, meta-analyses, letter to the editor, conference posters or abstracts, and purely technical feasibility studies.

### 2.4. Data Extraction

Data extraction was performed by 2 researchers (J.Q. and B.I.) and included first author, institution and year of publication. Key data points collected included population characteristics (age, sex, healthy or disease states), 4D-flow-derived novel flow parameters, key findings and conclusions, imaging analysis tools used, and any validation, internal consistency and reproducibility analyses. Furthermore, 4D-flow MR pulse sequence parameters were also collected from each study.

### 2.5. Quality Assessment

Quality assessment was performed by J.Q. and B.I. using a modified version of the Critical Appraisal Skills Program (CASP) tools which focused on evaluation of the clinical reproducibility based on the completeness of methodologies reported and the clinical relevance of the study outcomes rather than criticism of the validity of the methodologies presented [12]. Answers of “yes” scored 1 point, answers of “no” scored 0 points. A percentage of the maximum available score was calculated, and studies were allocated to one of three categories based on their percentage: highly clinically applicable (67–100%), potentially clinically applicable (34–66%), less clinically applicable (0–33%) [12].

### 2.6. Quantitative Assessment

Due to the heterogeneity in study design and outcome, a generalised meta-analysis was not possible for this systematic review. However, analysis of similarities in reported outcomes was performed, and a narrative review is provided.

## 3. Results

### 3.1. Search Strategy

**Figure 1:**
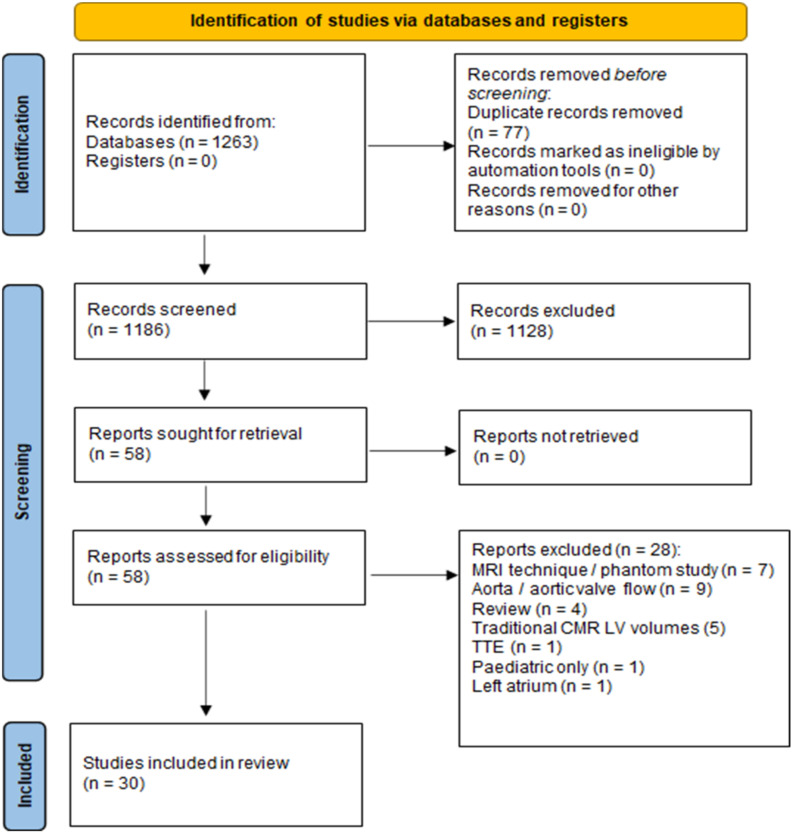
PRISMA flow diagram showing studies inclusion process [11].

An initial search in the databases yielded 1263 articles. After removal of duplicates, 1186 articles remained. Based on eligibility criteria, screening of title and abstract was performed, resulting in 58 articles remaining. Further evaluation of these articles through review of the full-text manuscript excluded a further 28 articles, resulting in 30 articles for inclusion based on selection criteria (Figure 1).

### 3.2. Description of the Included Studies

A total of 30 studies were included in the final review with a total of 1070 participants. A detailed summary of the studies is provided in Table S1 (Supplementary Materials). There was 100% agreement between observer assessment of clinical applicability, with all included studies ranked as “highly clinically applicable” by both investigators. All of the studies were observational studies, with one case-controlled study [13], one retrospective study [14], and the remaining 28 were cross-sectional studies. Ten studies were conducted with healthy volunteers only, and one study compared pre- and post-dobutamine stimulation in a healthy cohort. Two studies compared elite athletes with sedentary healthy controls. Four studies included patients with ischaemic heart disease. One study included patients following surgical correction of atrioventricular septal defect (AVSD). The remaining 12 studies included patients with heart failure secondary to various aetiologies.

### 3.3. Study Population

Participant demographics were variable across studies. The lowest participant number per study was 9 and the highest was 108 participants (total participants across all studies = 1070). The range of reported age mean was 24–71 years (standard deviation of reported means 16 years). For studies with a comparator cohort, three studies had statistical differences in age between comparator and control cohorts. One study with statistical differences in age was investigating the effect of ageing on LV function [15], another study had the control cohort drawn from a previously recruited population for another study [16], the third study had age-matched controls but also included a second younger control cohort [17].

### 3.4. 4D-Flow MR Pulse Sequence Parameters

There was considerable variability in the reported values of the 4D-flow sequence parameters and in the parameters reported between studies, these are detailed in Table S2 (Supplementary Materials). Reported spatial resolution ranged between 1.25–8 mm, and temporal resolution ranged between 35–90 ms. Echo time (TE) ranged between 1.9–6 ms, repetition time (TR) ranged between 3.4–68 ms, flip angle 5–15°, velocity encoding (VENC) 60–250 cm/s. Scan time was reported to range between 5–57 min; however, this was inconsistently reported as scan time for 4D-flow sequence only, total scan time including other sequences, or including or excluding respiratory gating efficiency. The majority of the studies had retrospective electrocardiogram (ECG) gating and navigator respiratory gating. Ten studies performed 4D-flow acquisition following a single bolus injection of gadolinium contrast, while the remainder of the studies did not specifically report on the use of contrast.

### 3.5. Image Analysis Tools and Methodologies

Image analysis tools were variable across studies, with many studies utilising multiple platforms for analysis. For segmentation of LV contour, Segment (Medviso, Lund, Sweden) was the most commonly used commercially available software. Several studies also reported the use of in-house modules within Segment. For flow and flow parameter visualisation, Ensight (CEI Inc., Research Triangle Park, NC, USA) was commonly used (eight studies). Other flow visualisation software utilised included GTFlow (one study; GyroTools, Zurich, Switzerland), Paraview (one study; Kitware, Clifton, NY, USA), and iTFlow (two studies; Cardio Flow Design Inc., Tokyo, Japan). MATLAB (11 studies; Mathworks, Natick, MA, USA) was also commonly used for computation of flow parameters. One research group used an in-house software MASS (Leiden University Medical Center, Leiden, The Netherlands) to perform their analysis. Prior to analysis, images were corrected for aliasing, phasing offsets and errors using either scanner or in-house algorithms. Where reported, segmentation of LV contour was performed manually or semi-manually using 2D cine data on a specialised platform, then registered with 4D-flow data manually or with dedicated software. Further quantification, analysis and visualisation were then performed on one or more additional software. Analysis time was generally not reported, although one study reported a total post-processing time of around 60 min per subject [18].

### 3.6. Scan–Rescan Reproducibility

Scan–rescan reproducibility was the primary aim of two studies [19,20,21] and was reported as a secondary analysis in a third study [21]. In Kamphuis et al. [19], twelve volunteers underwent two 4D-flow MRI scans 10 min apart, with repositioning and replanning performed for the second scan. Reproducibility was analysed for kinetic energy (KE), energy loss (EL) and vorticity. Reproducibility was good to strong for KE and EL but with a wide range of agreement (intraclass correlation (ICC): 0.64–0.95, coefficients of variation (CV) ≤ 25%). Reproducibility was good to excellent for vorticity (ICCs: 0.83–0.95, CVs ≤ 11%). Stoll et al. [20] evaluated the measurement reproducibility of LV flow components and the KE of each component in 45 healthy subjects. Ten subjects underwent two scans on the same day (“scan–rescan”) while 25 different subjects underwent the second scan at least 10 days after the first scan (“interval scan”, median 52 days, interquartile range (IQR) 28–57 days). The scan–rescan reproducibility was good for flow components volume ratio (CV: 2.5–9.2%) but was more variable for KE (CV: 13.5–17.7%), with the direct flow component having the lowest and delayed ejection flow having the highest variation in both volume ratios and KE. The interval scan reproducibility had greater variability than the scan–rescan cohort for both flow components volume ratio (CV: 6.2–16.1%) and KE (CV: 16.9–29.0%). In this cohort, direct flow had the lowest variation for both volume ratio and KE, but retained flow and residual volume had the highest variation for volume ratio and KE, respectively. The variations observed especially in the interval scan cohort were attributed to normal physiological variations, especially heart rate, but also fluid status, vascular tone and hormonal status. The CV range reported for KE was within the same magnitude reported by Kamphuis et al. [19]. Carlsson et al. [21] attempted to quantify KE in the LV and RV over the entire cardiac cycle in nine healthy individuals. Six individuals were imaged in both a 1.5 T and a 3 T scanner on the same day. Bias in energy peak between the two scanners was 0.58 ± 1.28 mJ for the six individuals, which was similar to the bias reported in Kamphuis et al. (0.1 ± 2.3 mJ). Outliers were only commented on in one study [19], where two subjects were found to be outliers with significantly higher variability between scans, which was attributed to possible physiological variations between scans.

### 3.7. Intra-/Inter-Observer Reproducibility

Intra- or inter-observer reproducibility analysis was mentioned or specifically reported in 11 studies. One study [22] briefly mentioned in the manuscript narrative that qualitative intra-/inter-observer reproducibility assessment of the image segmentation process was performed but did not report specific methodology or outcome of the reproducibility assessment. Reproducibility of measurement of the flow parameters investigated in this study was not reported. The remaining 10 studies all reported using standard statistical methodologies (e.g., Bland–Altman analysis, paired *t* tests) and presented the results of their analysis on the 4D-flow derived LV parameters investigated in their studies. Three studies performed reproducibility analysis on KE [17,23,24], two reported on LV flow components [20,25], two reported on vortex core features [18,26], one on ventricular residence time distribution [3], one on LV vorticity [27], one on qualitative assessment of streamline but not on EL which was also investigated in the study [14]. Good intra- and/or inter-observer reproducibility was reported in all studies that performed the analyses (Table 1).

### 3.8. Validation Methods

Validation of 4D-flow derived flow parameters was specifically reported in only one study through the use of a separate phantom experiment performed using the same methodology [28]. In this study, KE was evaluated in the four heart chambers in athletes and sedentary healthy individuals. KE was validated using a phantom setup consisting of a water tank for vortex ring generation and a water pump. Velocities were then measured using 4D-flow and particle imaging velocimetry (PIV). Downsampling of measured data was also performed to assess effects of reduced temporal and spatial resolutions. Very high correlation was found between KEs measured by 4D-flow and PIV (R^2^ = 0.99, mean ± 2SD: −0.02 ± 0.12 mJ). Reduced temporal and spatial resolutions both resulted in lower KE peak using both methods (by approx. 1–2 mJ). Mitral E/A ratio is a well-established parameter for quantification of diastolic function [29] and was evaluated in one study for comparison between two-dimensional phase-contrast (2D-PC) and 4D-flow parameters [23]. While not reported specifically as validation, the authors compared 4D-flow measured diastolic KE_iEDV_ (KE indexed to EDV) with 2D-PC measured mitral E/A ratio and found statistically significant positive correlation between the two parameters (r = 0.77, *p* < 0.01), suggesting the validity of the 4D-flow derived parameter for quantifying diastolic function [23].

### 3.9. Novel Flow Parameters

Novel LV flow parameters investigated by the included studies were ventricular kinetic energy (12 studies), LV flow components (5 studies), LV vortex morphology (4 studies), LV vorticity (4 studies), viscous energy loss (4 studies), LV haemodynamic forces (4 studies), residence time distribution (1 study), and turbulent KE (1 study). Table 2 summarises the flow parameters and their clinical applications in the studies.

#### 3.9.1. Ventricular Kinetic Energy

Transient kinetic energy (KE) or kinetic energy due to the time-varying components of the flow velocity is the work performed on blood in order to accelerate it from stationary to a specific speed. The transition in the flow velocity is associated with energy dissipation of the whole blood circulation; therefore, KE can be seen as a measure of blood flow efficiency. It has been suggested that by quantifying KE within the left ventricle, subtle and early changes in cardiac function may be detected prior to any clinical symptoms or obvious ventricular remodelling or dysfunction that is detectable by conventional means, potentially benefiting early diagnosis and intervention [2]. LV KE can be calculated using velocity measurements obtained from 4D-flow MRI by summation of KE of each voxel element over the total number of voxels covering the LV [24]:

(1) $$\mathrm{KE}=\frac{1}{2}\sum_{i=1}^{n}\rho \nu^{2}V_{n},$$

where ρ is blood density, ν is the blood velocity vector, $V_{n}$ is the *n*-th voxel volume. Reporting of KE in literature is heterogeneous, with outcomes reporting variably as raw values, or indexed to various LV parameters including EDV, SV and LV mass, or to body surface area (BSA). KE has been investigated in normal physiological ageing [15,23], ischaemic heart disease and heart failure [13,17,24,30], different sex [31,32], athletes [28], and healthy individuals [19,21,22].

Two studies reported LV KE measurements for ageing. Ageing has been associated with decreasing LV compliance, which in turn has an important role in ventricular filling efficiency [21,33]. Both studies found a decrease in KE relating to ageing, especially during early diastolic filling. Wong et al. [15] quantified LV KE in 35 healthy adults and children (age 1 to 67 years) and 10 adults with LV dysfunction (age 28–79 years). In the healthy cohort, peak early diastolic KE indexed to instantaneous ventricular volume showed a negative correlation with age (R^2^ = 0.545, *p* < 0.0001), while peak systolic KE was very weakly positively correlated with age (R^2^ = 0.206, *p* = 0.007). Mean early diastolic KE was significantly different between age groups (*p* = 0.0001) but there was no significant difference in mean systolic KE. Peak early diastolic KE in the LV dysfunction cohort showed similar magnitude to older healthy adults (*p* = 0.254). KE indexed to LV mass was found to be distinctively different between either end of the LV mass spectrum, suggesting a potential confounding effect on KE measurements of different heart sizes. Similar association between diastolic KE and age was found in 53 healthy adults aged 45 ± 17 years by Crandon et al. [23], who reported diastolic KE indexed to LVEDV (KE_iEDV_), found that increasing age resulted in statistically significant decline in peak E-wave KE_iEDV_ while peak A-wave KE_iEDV_ increased, resulting in declining KE_iEDV_ E/A ratio with increasing age (*p* < 0.05). Importantly, the 4D-flow measured KE_iEDV_ E/A ratio showed good correlation with the 2D mitral inflow E/A ratio (r = 0.77, *p* < 0.01). There was no association between systolic KE_iEDV_ and age. When reported as raw values (i.e., without indexation to any LV volume), there was no statistically significant age-related difference in any of the reported KE parameters.

Ischaemic heart disease is highly prevalent globally and carries significant mortality and morbidity [34]. LV remodelling secondary to acute myocardial infarction (AMI) or chronic ischaemic heart disease plays an important role in LV haemodynamics and overall prognosis [24]. Whilst regional wall motion, LV size and LVEF can be characterised with conventional methods, these metrics may not correlate adequately with clinical compensation and prognostication [35,36]. KE has been proposed in four studies as a novel method to quantify cardiac function in patients with ischaemic heart disease. The studies found reduced early diastolic KE in patients, which potentially contributed to the formation of LV thrombus (LVT). Findings were less consistent with late diastolic or systolic KE. Garg et al. [24] examined 48 patients (22 post-AMI, 26 with chronic ischaemic heart disease) against 20 healthy controls. Global, systolic and diastolic peak E-wave KE_iEDV_ were all found to be significantly lower in patients compared to controls (*p* = 0.02, *p* < 0.01, *p* = 0.02 respectively). Peak A-wave KE_iEDV_ was not different between the two groups (*p* = 0.22). In addition, systolic KE_iEDV_ decreased significantly with decreasing LVEF. Time difference (TD) to peak E-wave KE was significantly higher in the patient group (*p* < 0.01). Differences in KE were also observed between patients with preserved LVEF and controls, suggesting the presence of cardiac dysfunction despite apparently normal LVEF. In a separate study, Garg et al. [17] quantified KE in post-MI patients with or without LVT and compared against age-matched and younger controls. The most significant finding was the delayed wash-in of LV during diastole as quantified by TD of peak E-wave KE propagation from LV base to mid-cavity which increased from healthy controls to patients without LVT, and further increased from patients without LVT to those with LVT (overall ANOVA between groups <0.001). No difference in TD was observed between younger and aged-matched controls (*p* = 0.52). Furthermore, in-plane KE as a proportion of total KE was higher in patients with LVT than those without (*p* = 0.002), further supporting the notion that patients with LVT had reduced through-plane flow hence global wash-in. Reduced through-plane flow in post-MI patients was also identified by Corrado et al. [13]. Their study did not find a difference in KE_iEDV_ between patients and healthy controls; however, only the KE_iEDV_ averaged over the entire cardiac cycle was reported, while differences in KE_iEDV_ were identified at specific cardiac phases by the other two studies. In Kanski et al. [30], KE was quantified in patients with ischaemic heart disease with LVEF < 40% and healthy controls. Systolic averaged KE_iEDV_ was found to be lower in patient cohorts (*p* = 0.025) but no difference between patients and controls in diastolic averaged KE_iEDV_ (*p* = 0.41). When indexed to SV, both systolic- and diastolic-averaged KE_iSV_ were higher in patients compared to the control (*p* < 0.0001).

Other usage of KE has been in comparing diastolic function between elite athletes and healthy but sedentary individuals (higher early diastolic KE reported as raw values in athletes, *p* = 0.04 likely associated with larger LV mass) [28]. Studies comparing cardiac function between healthy males and females were less consistent. One study reported higher peak systolic KE in males than females (*p* = 0.047) when reported as a raw value, but when indexed to SV, the difference was not significant (*p* = 0.353) [31]. A second study by the same research group found statistically significantly higher systolic and diastolic KE_iSV_ in males than females (*p* = 0.04, *p* = 0.07 respectively), but no difference when KE was indexed to EDV [32]. Potential reasons for the discrepancies included inadequate sample size, physiological variations between studies, and potential confounding effects of different heart sizes and volumes, making some KE metrics less suitable for reporting.

#### 3.9.2. Flow Components

LV flow components were first identified and delineated using 4D-flow MRI and particle trace analysis by Bolger et al. [37] in 17 healthy individuals. By tracing the flow paths of imaginary particles through the velocity field acquired by 4D-flow MRI within the LV, pathlines were created to visualise the movement of blood over a cardiac cycle. Four components of LV flow were identified as direct flow (DF), retained inflow (RI), delayed ejection flow (DE), and residual volume (RV), with DF and DE constituting ejecting volume, and RI and RV the non-ejecting volume. KE of each component can also be calculated by integration over the path lengths. DF was found to be the most efficient component with the shortest path length from mitral orifice to the LV outflow tract (LVOT) while retaining most of its KE, thus requiring the least LV energy for ejection. Alterations in the relative makeups of the flow components as well as their KEs have since been used to quantify ischaemic heart disease and heart failure [8,13], LV dyssynchrony [38], and the effect of dobutamine on cardiac efficiency in healthy subjects [25].

DF component was the most impacted in patients with ischaemic heart disease with or without reduced systolic function and LV remodelling. Svalbring et al. [8] attempted to use flow components to detect subtle LV remodelling in a study involving 26 patients with no to mild LV remodelling or reduced systolic function and no more than New York Heart Association (NYHA) Class II symptoms, with 10 healthy subjects. The volume of all four components increased with LVEDV (r = 0.64, 0.77, 0.75 and 0.86 for DF, DE, RI and RV, respectively; *p* < 0.005 for all four comparisons). Proportion of DF relative to LVEDV decreased with increased LVEDV index (LVEDVI) and LVESV index (LVESVI). Proportions of DF and non-ejecting components KEs relative to total EDV KE at end diastole decreased and increased, respectively, with increased LVEDVI and LVESVI. LVEF was positively correlated with DF volume and KE (r = 0.68, *p* < 0.001 and r = 0.47, *p* < 0.05, respectively), while negatively correlated with non-ejecting volume and KE (r = −0.74, *p* < 0.001 and r = −0.44, *p* < 0.05, respectively). Similarly, Corrado et al. [13] found in patients post-AMI with reduced EF vs. healthy controls, DF volume as a proportion of total flow volume was significantly lower in the patient cohort, while all the other flow components were higher compared to controls (DF: 26% vs. 58%, *p* = 4 × 10^−8^; RI: 24% vs. 15%, *p* = 0.0003; DE: 18% vs. 16%, *p* = 0.005; RV: 29% vs. 7%, *p* = 6 × 10^−6^).

Heart failure with left bundle branch block (LBBB) results in dyssynchronous LV contraction and relaxation and is associated with higher mortality and morbidities. Cardiac resynchronisation therapy (CRT) is a potentially beneficial therapy with proven mortality benefits in heart failure patients with LBBB, but a significant proportion of patients remain non-responders [39,40]. Zajac et al. [38] proposed flow components as potential functional biomarkers for better characterisation of LV dyssynchrony that may also predict response to CRT. The study included 22 patients with or without LBBB (50:50 split), and quantified LV flow by its four components, and for each component quantified contribution from E- and A-filling. The study found that while there was no difference in the volume of each flow component between the two groups, the end diastolic KE of DF overall and the E-filling component of DF was lower in the LBBB group (*p* = 0.008, *p* = 0.017 respectively). There was no difference between groups in the end diastolic KE of the A-filling component of DF and the remaining flow components. The findings suggested that LV dyssynchrony impacted on early diastolic filling, while late diastolic filling was less affected due to reliance on atrial contraction. The authors speculated that impaired DF KE could potentially be investigated as a predictor of responsiveness to CRT. Sundin et al. [25] investigated the effect of dobutamine in 12 healthy subjects and found that DF as a proportion of LVEDV increased by 16% (*p* < 0.001) post-dobutamine administration, while DE and RV reduced by 4% (*p* < 0.001) and 11% (*p* < 0.001) respectively, and RI had no significant change (*p* = 0.43). However, KE_iEDV_ at end-diastole increased for all four flow components (DF from 7.7 ± 3.0 to 21.0 ± 5.4 μJ/mL; RI from 3.7 ± 1.4 to 9.6 ± 3.1 μJ/mL; DE from 5.8 ± 2.5 to 13.6 ± 6.0 μJ/mL; RV from 1.5 ± 0.5 to 2.8 ± 1.0 μJ/mL; *p* < 0.001 for all four components). Overall, there appears to be consistency between studies on finding the impact of LV function on DF volume proportional to LVEDV and flow component KE.

#### 3.9.3. LV Vortex Morphology

**Figure 2:**
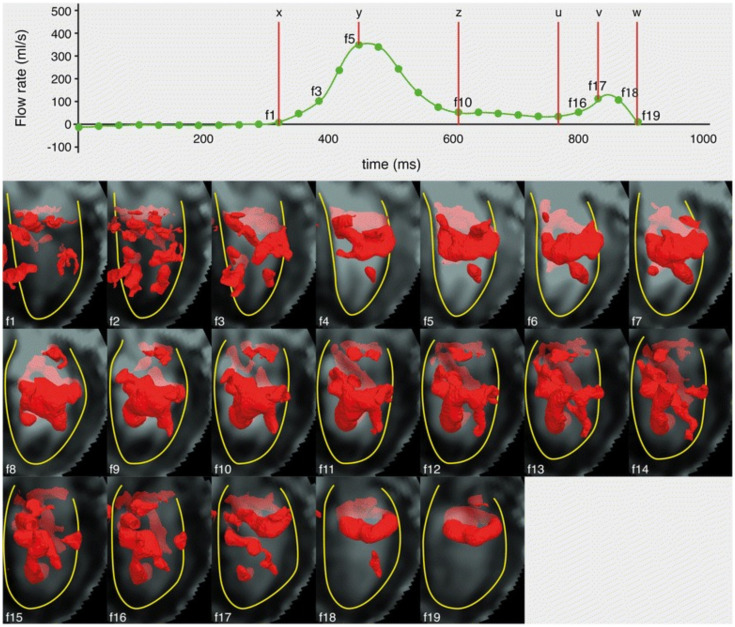
LV vortex structures visualised as isosurfaces in red colour using the Lambda2 method over the diastolic phase of a sample normal subject (**f1**ߝ**19**), with E-filling onset (x), peak (y) and end (z), and A-filling onset (u), peak (v), and end (w) marked on the flow rate time curve (top panel). The compact vortex ring formed between early (**f3**) and late (**f7**) diastolic filling became most developed during early- (**f5**) and A-filling (**f18**), and continued to the end of late filling (**f19**) [26]. Permission to reproduce obtained under the Creative Commons Public Domain Dedication waiver via Springer Nature.

A vortex is a 3D structure where fluid particles rotate about a common axis [26]. Concentrated vortices are observed in many natural phenomena, and a frequently observed type of concentrated vortex is the vortex ring, which is a tube-like vortical structure closed in a ring moving in isolation to the surrounding fluid known for its compactness and stability [26,41,42]. LV diastolic filling vortex ring is a well-recognised structure which is thought to contribute critically to blood pumping efficiency [43]. Furthermore, 4D-flow MRI has been proposed as a suitable modality to characterise this complex, dynamic 3D structure in the LV. In 24 healthy subjects, Elbaz et al. [26] identified two distinctly separate vortex rings associated with E-filling and A-filling. Both vortex rings were compact and quasi-torus shaped and related closely to the mitral valve leaflets with the A-filling ring located closer to the valve. Orientations of the two vortex rings in relation to the LV axes were similar. E-filling is associated with the passive diastolic filling of the LV due to ventricular relaxation, while A-filling is associated with the contraction of the atrium in late diastole (Figure 2). Abnormalities of E- and A-filling are markers of diastolic dysfunction [44], which may be reflected in abnormal vortex formation. Calkoen et al. [45] found that mitral valve morphology impacted on vortex formation. In 32 patients with surgically corrected AVSD, 26 patients had a compact albeit more deformed E-filling vortex and similar vortex formation time (VFT) compared to healthy controls, while 6 patients did not have an E-filling vortex. These patients had altered or smaller mitral valve opening, higher peak velocity through valve and prolonged VFT compared to healthy control, suggesting that surgical approach and impact on mitral valve morphology during AVSD repair could potentially impact on diastolic filling efficiency. Suwa et al. [18] analysed vortex morphology comparing patients with preserved and impairment LV function. In LV with preserved function, compact vortex rings were again identified located close to the mitral valve leaflets during both E- and A-filling without continuation between the two phases. No vortex was seen during systole. In the setting of impaired LV function, the diastolic vortex formed more distally to the mitral valve towards the apex and was larger and less compact. The vortex formed during E-filling and continued throughout diastole without dissipation. In addition, 57% of the patients exhibited a systolic vortex. Distance to vortex core (from atrio-ventricular junction) and vortex area both correlated strongly with LVEDV (r = 0.66 and 0.73, *p* < 0.01), LVEF (r = −0.74 and −0.68, *p* < 0.01), LV sphericity index (r = −0.60 and −0.65, *p* < 0.01), and peak filling rate (r = −0.61 and −0.64, *p* < 0.01). Miyajima et al. [11] found that in the presence of LBBB vortex rotational direction was reversed and was associated with higher energy loss, potentially indicating less efficient LV haemodynamics from non-physiological vortex rotation due to LV dyssynchrony.

#### 3.9.4. LV Vorticity

Vorticity is the measurement of the tendency for a fluid to rotate. In two studies, Schäfer et al. [27,46] investigated the potential of LV vorticity as a biomarker for diastolic dysfunction. Both studies found that peak E-wave vorticity was significantly lower in the patient cohort but with no difference in peak A-wave vorticity between patients and controls. In one study with patients with pulmonary hypertension [27], E-wave vorticity was correlated with LV eccentricity index, and diastolic vortex was absent in patients, suggesting an interventricular interdependence between LV and RV, and reduced LV vorticity and vortex formation due to septal deviation. No correlation was found between E-wave vorticity and LV chamber size, volumes or cardiac output. In the other study with patients with chronic pulmonary obstructive disease (COPD) [46], E-wave vorticity was lower in patients compared to controls even in the absence of apparent diastolic dysfunction (DD) as assessed by TTE (COPD with LVDD vs. COPD without LVDD vs. control: 2419 vs. 4075 vs. 7891, *p* < 0.0001 for both patient cohorts comparison with controls), suggesting the potential of vorticity in diagnosing subclinical LV dysfunction. In healthy subjects, Pewowaruk et al. [32] and Rutkowski et al. [31] both found that healthy females had higher LV vorticity than males; however, they did not appear to influence cardiac output or energy transfer from LV to the aortic outflow tract.

#### 3.9.5. Viscous Energy Loss

Viscous energy loss (EL) is the loss of mechanical energy mainly to thermal energy due to fluid viscosity and friction. The preservation of energy within the LV is thought to be indicative of blood pumping efficiency [47]. In Elbaz et al. [47], two peaks of EL_iSV_ were identified during diastole which correlated with E-filling and A-filling peaks of flow rate and KE. Patients with corrected AVSD had significantly higher EL_iSV_ than healthy controls during peak and average E- and A-filling (*p* < 0.001). Patients with higher EL_iSV_ also had an abnormally oriented diastolic vortex ring, while vortex ring orientations were similar between patients and controls with similar EL_iSV_. The highest EL_iSV_ was found in patients without a vortex ring, highlighting the role of diastolic vortex in LV diastolic efficiency. Miyajima et al. [14] also found higher EL in patients with LBBB than those without LBBB, again suggesting the impact of LV dyssynchrony on cardiac efficiency. The role of diastolic vortex in preserving LV energy was also evaluated in Nakaj et al. [22]. The vortex in healthy subjects appeared to facilitate smooth ejection of blood out of the LV with minimal change in EL_iBSA_. In addition, there appeared to be a statistically significant but weak to moderate association between RV SV and LV EL (r = 0.4795, *p* = 0.040), suggesting that RV plays a role in regulating systemic blood flow energy dynamics. However, this association was not found with LV EL_iBSA_, highlighting the uncertainty around reporting of the metric. Pewowaruk et al. [32] did not find significant differences in EL_iSV_ or EL_iEDV_ between healthy females and males (*p* = 0.24 and 0.13 respectively).

#### 3.9.6. LV Haemodynamic Forces

Haemodynamic forces are exchanged between the myocardium and blood which drive intracardiac blood flow. LV haemodynamic forces are computed by integrating the pressure gradient over ventricular volume [48]. Direction and the degree of dispersion of haemodynamic forces appeared to be important metrics. Eriksson et al. [49] found that in patients with dilated cardiomyopathy (DCM), distribution of force directions measured by force ratio (short-axis max force/long-axis max force) were greater compared to healthy controls in both early and late diastolic filling (*p* < 0.0001, *p* < 0.03, respectively). Force ratio during early diastolic filling was also higher when comparing DCM patients with apparently normal diastolic function and healthy controls (*p* < 0.0001), but not during late diastolic filling (*p* = 0.0903). Similar findings were also reported in a separate study by Eriksson et al. and by Arvidsson et al., and both found early diastolic force ratio to be higher in patients with LBBB compared either to patients without LBBB [50] or healthy controls [16]. The findings suggested that in a pathological LV, greater proportion of the haemodynamic forces were distributed transversely orthogonal to the main flow directions in the ventricle, reducing LV pumping efficiency. Heart size apparently had no impact on haemodynamic forces as investigated by Arvidsson et al. [48] in athletes and non-athletic healthy subjects.

#### 3.9.7. Residence Time Distribution

Costello et al. [3] proposed residence time distribution (RTD) as a novel metric for ventricular function. RTD is the cumulative distribution of time it takes for blood to transit through a heart chamber and exit. It is routinely used in chemical reactors to evaluate mixing and flow efficiency. In 32 patients with dilated cardiomyopathy and healthy subjects, RTD was found to be significantly higher and more variable in patients compared to controls (2.2 ± 0.80 vs. 1.2 ± 0.13, *p* < 0.001). There was also strong correlation between RTD and LVEF (R = −0.843, *p* < 0.001) across both patients and controls, and strong correlation between RTD and global longitudinal strain (R = 0.786, *p* < 0.001), suggesting a strong relationship between myocardial deformation and blood flow efficiency. The authors suggested that RTD may have diagnostic values in early LV dysfunction but will require further evaluation.

#### 3.9.8. Turbulent Kinetic Energy

Turbulent kinetic energy (TKE) is the kinetic energy that counts for variations in the magnitude of the blood flow velocity, where the flow’s viscous forces dominate over inertial forces and lead to the appearance of vortices, resulting in the dissipation of kinetic energy and drop in pressure gradient. High values of TKE can be interpreted as an indication of blood flow abnormalities which can cause regional or global adverse effects on the cardiovascular system [51]. Zajac et al. [51] found that in patients with dilated cardiomyopathy, peak TKE was significantly higher during late diastolic filling compared to healthy controls (3.0 ± 1.8 vs. 1.5 ± 0.8 mJ, *p* = 0.02). However, there was no difference in peak TKE between groups during early diastolic filling. LV diameter during diastole was greater (*p* < 0.001), and mitral annular diameter was larger during late diastolic filling (*p* = 0.09) but not early diastolic filling (*p* = 0.35) in DCM patients compared to control. The reason for TKE differences between patients and controls only being observed during late diastolic filling was suggested to be due to higher or rising LV pressure from greater retained flow and abnormal vortex formation during late diastole in DCM patients.

## 4. Discussion

In this systematic review, we analysed current literature using 4D-flow MRI-derived flow parameters to quantify LV function. To the best of our knowledge, this is the first review which systematically summarised the use of 4D-flow derived parameters for the quantification of LV function with a clinical focus [2,7,12,52,53,54]. Overall, we found that all studies that met our criteria were small-scale, cross-sectional feasibility or pilot studies establishing the potential of the flow parameters. The large amount of heterogeneity between studies greatly limited quantitative inter-study comparison, limiting the evaluation of the readiness of any of the flow parameters for clinical application. The main findings of the review are: (1) the generalisation of 4D-flow MRI in clinical applications has been limited by the lack of standardisation in protocol, methodology and analysis approaches, which also limited the inter-study assessment of clinical applicability of 4D-flow derived flow parameters; (2) energy related flow parameters, flow components and LV vortex were most studied and showed potential in detecting early diastolic dysfunction or subtle LV remodelling; (3) other flow parameters remain in early exploratory stage and will require further studies to evaluate their clinical utilities.

A major factor currently limiting the broader clinical use of 4D-flow MRI is the lack of standardisation in protocol, methodology and analysis [10,55], which is consistent with our findings. The pulse sequence parameters and resultant image resolutions were heterogeneous across the studies but were within recommended parameters [56], likely due to differences in scanner types, magnet strength, acceleration methods, and local experience (Table S2, Supplementary Materials), and it was not possible to directly compare the clinical impact of these differences between studies. Image analysis tools were various with many in-house modules and methodologies that would be difficult to generalise or translate. Quantification of intraventricular flow is challenging, and the process of segmentation, image registration, visualisation and quantification is typically onerous and requires considerable training and expertise. Each of these steps entails considerable detail, which is often not fully reported, and variations in analysis or measurement approach could lead to a large variance in measurements. Homogenisation of acquisition and processing are clearly key for future translation in this space [55].

Reporting of reproducibility was variable, although some studies referenced previous works where intra- or inter-observer reproducibility was performed on conventional MR parameters. Scan–rescan reproducibility is considerable especially for KE (CV up to 29%), with differences attributed primarily to physiological variations. There is a lack of validation of novel flow parameters; however, this is difficult in view of the lack of appropriate gold standard comparators. Another limiting factor for clinical applicability is the long scan time. Despite most studies reporting the use of parallel imaging and acceleration methods, the range of reported scan times was mostly 5–25 min, with one outlier study reporting scan time up to 57 min [49]. The upper end of this range may still be too long for some clinical settings.

Many novel flow parameters have been proposed, amongst which KE appeared to be the most studied. While there was overall consistency in findings between studies, it was not possible to quantitatively compare the results due to significant heterogeneity in study design, subject demographics and metrics reported. KE was reported variably as raw values, or indexed to LV volumes, LV mass or to flow component volumes, and as peak or averaged values, with occasionally confounding results between the metrics. As a result, it remains unclear which is the most suitable and clinically applicable method for reporting, although KE_iEDV_ measured at peak E- and A-filling appeared to show most consistency based on the included studies. Other energy-related metrics (EL and TKE) also showed promise but will require further evaluation to demonstrate their standalone values. Based on the reported findings in relevant studies, each parameter may only be suitable for quantification of certain cardiac phases. For example, there was no difference found in TKE between patients and controls during early diastolic filling, while significant difference in EL was found across the whole diastolic phase. Significant differences in KE between patients and controls were also primarily found in early diastolic filling and were less consistently in late diastolic filling and systole. Flow components analysis is a novel method for quantitative assessment of LV diastolic function, and other parameters such as KE can be computed on each component to provide further insights. While there appears to be consistency between studies on flow components analysis, the small sample size limited their study power. Further splitting of flow components may also be useful in regional flow analysis. Similarly, LV vortex morphology is a promising qualitative and quantitative method which has the potential to provide insight into flow behaviour in healthy and pathological states as well as interaction between blood flow and anatomical structures including the mitral valve, which may have implications on future surgical approaches. Limited studies exist for vorticity, haemodynamic forces and RTD, and these parameters will require further investigation to assess clinical relevance and applicability.

Furthermore, 4D-flow MRI remains a relatively new technique, and the use of 4D-flow MRI-derived flow parameters to evaluate the LV is still in an exploratory phase. Based on existing literature, it remains unclear how these flow parameters can be best applied in clinical applications. Future directions of research will likely need to involve larger-scale, prospective studies to further validate the diagnostic values of each novel parameter. To enable this, the development and standardisation of acquisition and analysis protocols, methodologies and software tools will be required. A large-scale, multi-centre trial (4D-flow MRI for Cardiovascular Evaluation (4DCarE), Australia and New Zealand Clinical Trials Registry number 382990, Universal Trial Number U1111-1270-6509) currently underway in our research group aims to address some of these aspirations. Furthermore, 4DCarE, with a target recruitment size of 800–1000 participants, aims to evaluate the non-inferiority of 3D (4D-flow and 3D-cine) to 2D (2D-phase contrast and short-axis cine) acquisition techniques in clinical evaluation of cardiac function and pathologies. The trial also aims to evaluate the feasibility of a standardised image analysis process using commercially available analysis software to improve cost-effectiveness and generalisation. Leveraging the size of the study cohort, the trial will also aim to investigate the clinical applicability of 4D-flow derived novel flow parameters through a series of sub-studies. Achieving the trial objectives will potentially make possible broader clinical application of 4D-flow acquisitions, reduced time, effort and cost of image acquisition and analysis, and broader systematic evaluation of novel flow parameters derived by 4D-flow.

## 5. Conclusions

In summary, 4D-flow MRI has great potential to provide invaluable insights into LV function, and many novel flow parameters have been proposed. Current relevant literature consists of small-scale feasibility or pilot studies with considerable heterogeneity in study design, methodology and data reporting. There remains considerable work to standardise image acquisition protocols and analysis methodologies, and larger-scale studies are required before any novel LV flow metrics can be translated to clinical applications.

## Figures and Tables

**Table 1:** Intra-/inter-observer reproducibility methodologies and results.

Study	Intra-/Inter-Observer Reproducibility Methodology	Reproducibility Results
Costello et al., 2018 [3]	ICC of LV and RV RTDc	Intra-observer: LV 0.901 (*p* < 0.001), RV 0.768 (*p* = 0.004); Inter-observer: LV 0.881 (*p* < 0.001), RV 0.728 (*p* = 0.008)
Crandon et al., 2018 [23]	CV; 10 cases for intra- and 20 cases for inter-observer	Average CV for all variables: 6 ± 2%; Intra-observer CV: global KE 3.5%, systolic KE 3.9%, diastolic KE 6.0%, peak E-wave KE 4.4%, peak A-wave KE 5.5%; Inter-observer CV: global KE 7%, systolic KE 11%, diastolic KE 6.4%, peak E-wave KE 6.6%, peak A-wave KE 6.3%
Elbaz et al., 2014 [26]	Intra-observer: repeat measurements by same observer one week apart; Inter-observer: two observers independently assessed same database; ICC of C, L, R coordinates and orientation of vortex ring cores	Intra-observer: ICC = 0.97, *p* < 0.001, CV 1–3%; Inter-observer: ICC = 0.96, *p* < 0.001, CV 1–8%
Garg et al., 2018 [24]	Inter-observer: ICC of KE computed from short-axis LV contours segmented by two observers independently; Intra-observer: ICC of KE computed from short axis LV contours segmented by same observer 3 months apart	Overall ICC for all global KE parameters: average 0.99, *p* > 0.9; TD to peak E-wave KE ICC = 0.94, 95% CI 0.88 to 0.97 Intra-observer: mean bias for KE_iEDV_ 3 ± 9%; Inter-observer: mean bias for KE_iEDV_ 2 ± 9%;
Garg et al., 2019 [17]	Inter-observer: ICC of KE computed from short-axis LV contours segmented by two observers independently; Intra-observer: ICC of KE computed from short axis LV contours segmented by same observer 3 months apart; Inter-rater reliability κ of KE and TD;	Intra-observer: global KE parameters bias 2%, precision −16%; Inter-observer: global KE parameters bias 4%, precision −20%; Inter-rater reliability weighted kappa: in-plane KE κ = 1, distal A-wave KE drop κ = 0.63, and TD from base to mid κ = 0.67
Miyajima et al., 2021 [14]	Inter-observer: κ of inflow pattern analysis	κ = 0.68
Nakaji et al., 2021 [22]	Qualitative assessment of segmentation process by radiologist, cardiac surgeon, masters student in more than 3 cases	Small qualitative differences
Schäfer et al., 2016 [27]	Inter-observer: ICC of LV vorticity	E-wave vorticity ICC = 0.94; A-wave vorticity ICC = 0.93
Stoll et al., 2018 [20]	Intra-observer: two blinded assessments by same observer one month apart; Inter-observer: two observers performed assessment on same dataset independently	Intra-observer: CV of flow components 3.6–6.1%; Inter-observer: CV of flow components: 2.6–5.7%
Sundin et al., 2020 [25]	ICC of LV flow component	Intra-observer: LV flow component with dobutamine ICC = 0.99; Inter-observe: LV flow component with dobutamine ICC = 0.80–0.91
Suwa et al., 2016 [18]	Inter-observer: κ or CV for the determination of the presence of an intra-LV vortex and vortex features	Determination of presence of vortex: κ = 0.867; Estimation of vortex features: CV distance to vortex core, 0.83; vortex area, 0.85; sphericity index of the vortex, 0.94; *p* < 0.01

CV: coefficient of variability; ICC: intra-class correlation coefficient; κ: Cohen’s weighted kappa; KE: kinetic energy; RTDc: residence time distribution constant; TD: time difference.

**Table 2:** Summary of flow parameters and clinical applications.

Novel Flow Parameter	Definition	Number of Studies	Clinical Applications	Current Limitations	Metrics Most Likely to Have Clinical Potential
Kinetic energy	Work performed on blood to accelerate it from stationary to a specific speed	12	Ageing; ischaemic heart disease; heart failure; sex differences; athletes	Reported variably as raw value or indexed values, and as averaged or at specific cardiac phases	KE indexed to LVEDV at peak E- and A-filling
Flow components	Blood components with different flow paths over a cardiac cycle	5	Ischaemic heart disease; heart failure; LV dyssynchrony; dobutamine stress	Reported variably as volume proportional to total flow volume or LVEDV; limited studies quantifying component KE	Flow component volume as a proportion of total flow volume or LVEDV; component KE indexed to LVEDV
Vortex morphology	Shape, location, orientation and dimension of the LV diastolic vortex	4	AVSD; heart failure; diastolic dysfunction; LV dyssynchrony	Limited studies; no standardised measurement approach	Vortex location relative to MV; vortex dimensions
Vorticity	Tendency for a fluid to rotate	4	Diastolic dysfunction associated with COPD and pulmonary hypertension; sex differences	Limited studies; uncertain clinical utility	Vorticity
Viscous energy loss	Loss of mechanical energy due to fluid viscosity and friction	4	AVSD; LV dyssynchrony	Limited studies; reported variably as indexed to SV, EDV or BSA; uncertain value as a standalone metric	EL indexed to SV, EDV or BSA
Haemodynamic forces	Forces exchanged between the myocardium and blood	4	Dilated cardiomyopathy; LV dyssynchrony	Limited studies; uncertain clinical utility	Haemodynamic force ratio
Residence time distribution	Cumulative distribution of time it takes for blood to transit through a heart chamber and exit	1	Dilated cardiomyopathy	Limited studies, uncertain clinical utility	Residence time distribution constant
Turbulent kinetic energy	Kinetic energy that counts for variations in the magnitude of blood flow velocity that leads to the appearance of vortices	1	Dilated cardiomyopathy	Limited studies, uncertain clinical utility	Turbulent kinetic energy

## Data Availability

Not applicable.

## Supplementary Materials

The following supporting information can be downloaded at: https://www.mdpi.com/article/10.3390/jcdd9090304/s1, Table S1—Study list, clinical applicability, population demographics, and quantitative parameters; Table S2—4D-flow MRI sequence parameters.

## References

[1] Burchfield J.S., Xie M., Hill J.A. (2013). Pathological ventricular remodeling: Mechanisms: Part 1 of 2. Circulation.

[2] Zhuang B., Sirajuddin A., Zhao S., Lu M. (2021). The role of 4D flow MRI for clinical applications in cardiovascular disease: Current status and future perspectives. Quant. Imaging Med. Surg..

[3] Costello B.T., Qadri M., Price B., Papapostolou S., Thompson M., Hare J.L., La Gerche A., Rudman M., Taylor A.J. (2018). The ventricular residence time distribution derived from 4D flow particle tracing: A novel marker of myocardial dysfunction. Int. J. Cardiovasc. Imaging.

[4] Dorosz J.L., Lezotte D.C., Weitzenkamp D.A., Allen L.A., Salcedo E.E. (2012). Performance of 3-dimensional echocardiography in measuring left ventricular volumes and ejection fraction: A systematic review and meta-analysis. J. Am. Coll. Cardiol..

[5] Ohyama H., Hosomi N., Takahashi T., Mizushige K., Osaka K., Kohno M., Koziol J.A. (2003). Comparison of magnetic resonance imaging and transesophageal echocardiography in detection of thrombus in the left atrial appendage. Stroke.

[6] Markl M., Geiger J., Arnold R., Stroh A., Damjanovic D., Föll D., Beyersdorf F. (2011). Comprehensive 4-dimensional magnetic resonance flow analysis after successful heart transplantation resolves controversial intraoperative findings and reveals complex hemodynamic alterations. Circulation.

[7] Azarine A., Garçon P., Stansal A., Canepa N., Angelopoulos G., Silvera S., Sidi D., Marteau V., Zins M. (2019). Four-dimensional Flow MRI: Principles and Cardiovascular Applications. Radiographics.

[8] Svalbring E., Fredriksson A., Eriksson J., Dyverfeldt P., Ebbers T., Bolger A.F., Engvall J., Carlhäll C.-J. (2016). Altered diastolic flow patterns and kinetic energy in subtle left ventricular remodeling and dysfunction detected by 4D flow MRI. PLoS ONE.

[9] Kamphuis V.P., Westenberg J.J.M., van der Palen R.L.F., Blom N.A., de Roos A., van der Geest R., Elbaz M.S.M., Roest A.A.W. (2017). Unravelling cardiovascular disease using four dimensional flow cardiovascular magnetic resonance. Int. J. Cardiovasc. Imaging.

[10] Plein S., Kozerke S. (2021). Are we there yet?. JACC Cardiovasc. Imaging.

[11] Page M.J., McKenzie J.E., Bossuyt P.M., Boutron I., Hoffmann T.C., Mulrow C.D., Shamseer L., Tetzlaff J.M., Akl E.A., Brennan S.E. (2021). The PRISMA 2020 statement: An updated guideline for reporting systematic reviews. BMJ.

[12] Elsayed A., Gilbert K., Scadeng M., Cowan B.R., Pushparajah K., Young A.A. (2021). Four-dimensional flow cardiovascular magnetic resonance in tetralogy of Fallot: A systematic review. J. Cardiovasc. Magn. Reson..

[13] Corrado P.A., Macdonald J.A., François C.J., Aggarwal N.R., Weinsaft J.W., Wieben O. (2019). Reduced regional flow in the left ventricle after anterior acute myocardial infarction: A case control study using 4D flow MRI. BMC Med. Imaging.

[14] Miyajima K., Urushida T., Ito K., Kin F., Okazaki A., Takashima Y., Watanabe T., Kawaguchi Y., Wakabayashi Y., Takehara Y. (2021). Four-dimensional flow magnetic resonance imaging visualizes reverse vortex pattern and energy loss increase in left bundle branch block. EP Europace.

[15] Wong J., Chabiniok R., deVecchi A., Dedieu N., Sammut E., Schaeffter T., Razavi R. (2016). Age-related changes in intraventricular kinetic energy: A physiological or pathological adaptation?. Am. J. Physiol. Heart Circ. Physiol..

[16] Arvidsson P.M., Töger J., Pedrizzetti G., Heiberg E., Borgquist R., Carlsson M., Arheden H. (2018). Hemodynamic forces using four-dimensional flow MRI: An independent biomarker of cardiac function in heart failure with left ventricular dyssynchrony?. Am. J. Physiol. Heart Circ. Physiol..

[17] Garg P., van der Geest R.J., Swoboda P.P., Crandon S., Fent G.J., Foley J.R.J., Dobson L.E., Al Musa T., Onciul S., Vijayan S. (2019). Left ventricular thrombus formation in myocardial infarction is associated with altered left ventricular blood flow energetics. Eur. Heart J. Cardiovasc. Imaging.

[18] Suwa K., Saitoh T., Takehara Y., Sano M., Saotome M., Urushida T., Katoh H., Satoh H., Sugiyama M., Wakayama T. (2016). Intra-left ventricular flow dynamics in patients with preserved and impaired left ventricular function: Analysis with 3D cine phase contrast MRI (4D-Flow). J. Magn. Reson. Imaging.

[19] Kamphuis V.P., Westenberg J.J.M., van der Palen R.L.F., van den Boogaard P.J., van der Geest R.J., de Roos A., Blom N.A., Roest A.A.W., Elbaz M.S.M. (2018). Scan-rescan reproducibility of diastolic left ventricular kinetic energy, viscous energy loss and vorticity assessment using 4D flow MRI: Analysis in healthy subjects. Int. J. Cardiovasc. Imaging.

[20] Stoll V.M., Loudon M., Eriksson J., Bissell M.M., Dyverfeldt P., Ebbers T., Myerson S.G., Neubauer S., Carlhäll C.-J., Hess A.T. (2018). Test-retest variability of left ventricular 4D flow cardiovascular magnetic resonance measurements in healthy subjects. J. Cardiovasc. Magn. Reson..

[21] Carlsson M., Heiberg E., Toger J., Arheden H. (2012). Quantification of left and right ventricular kinetic energy using four-dimensional intracardiac magnetic resonance imaging flow measurements. Am. J. Physiol. Heart Circ. Physiol..

[22] Nakaji K., Itatani K., Tamaki N., Morichi H., Nakanishi N., Takigami M., Yamagishi M., Yaku H., Yamada K. (2021). Assessment of biventricular hemodynamics and energy dynamics using lumen-tracking 4D flow MRI without contrast medium. J. Cardiol..

[23] Crandon S., Westenberg J.J.M., Swoboda P.P., Fent G.J., Foley J.R.J., Chew P.G., Brown L.A.E., Saunderson C., Al-Mohammad A., Greenwood J.P. (2018). Impact of Age and Diastolic Function on Novel, 4D flow CMR Biomarkers of Left Ventricular Blood Flow Kinetic Energy. Sci. Rep..

[24] Garg P., Crandon S., Swoboda P.P., Fent G.J., Foley J.R.J., Chew P.G., Brown L.A.E., Vijayan S., Hassell M.E.C.J., Nijveldt R. (2018). Left ventricular blood flow kinetic energy after myocardial infarction—Insights from 4D flow cardiovascular magnetic resonance. J. Cardiovasc. Magn. Reson..

[25] Sundin J., Engvall J., Nylander E., Ebbers T., Bolger A.F., Carlhäll C.-J. (2020). Improved efficiency of intraventricular blood flow transit under cardiac stress: A 4D flow dobutamine CMR study. Front. Cardiovasc. Med..

[26] Elbaz M.S.M., Calkoen E.E., Westenberg J.J.M., Lelieveldt B.P.F., Roest A.A.W., van der Geest R.J. (2014). Vortex flow during early and late left ventricular filling in normal subjects: Quantitative characterization using retrospectively-gated 4D flow cardiovascular magnetic resonance and three-dimensional vortex core analysis. J. Cardiovasc. Magn. Reson..

[27] Schäfer M., Browning J., Schroeder J.D., Shandas R., Kheyfets V.O., Buckner J.K., Hunter K.S., Hertzberg J.R., Fenster B.E. (2016). Vorticity is a marker of diastolic ventricular interdependency in pulmonary hypertension. Pulm. Circ..

[28] Steding-Ehrenborg K., Arvidsson P.M., Töger J., Rydberg M., Heiberg E., Carlsson M., Arheden H. (2016). Determinants of kinetic energy of blood flow in the four-chambered heart in athletes and sedentary controls. Am. J. Physiol. Heart Circ. Physiol..

[29] Park J.-H., Marwick T.H. (2011). Use and limitations of e/e’ to assess left ventricular filling pressure by echocardiography. J. Cardiovasc. Ultrasound.

[30] Kanski M., Arvidsson P.M., Töger J., Borgquist R., Heiberg E., Carlsson M., Arheden H. (2015). Left ventricular fluid kinetic energy time curves in heart failure from cardiovascular magnetic resonance 4D flow data. J. Cardiovasc. Magn. Reson..

[31] Rutkowski D.R., Barton G.P., François C.J., Aggarwal N., Roldán-Alzate A. (2020). Sex differences in cardiac flow dynamics of healthy volunteers. Radiol. Cardiothorac. Imaging.

[32] Pewowaruk R., Rutkowski D., Johnson C., Wolfinger A., Roldán-Alzate A. (2021). Assessment of sex differences in ventricular-vascular coupling of left ventricular and aortic flow derived from 4D flow MRI in healthy, young adults. J. Biomech..

[33] Fujimoto N., Hastings J.L., Bhella P.S., Shibata S., Gandhi N.K., Carrick-Ranson G., Palmer D., Levine B.D. (2012). Effect of ageing on left ventricular compliance and distensibility in healthy sedentary humans. J. Physiol..

[34] McManus D.D., Gore J., Yarzebski J., Spencer F., Lessard D., Goldberg R.J. (2011). Recent trends in the incidence, treatment, and outcomes of patients with STEMI and NSTEMI. Am. J. Med..

[35] Ugander M., Ekmehag B., Arheden H. (2008). The relationship between left ventricular ejection fraction and infarct size assessed by MRI. Scand. Cardiovasc. J..

[36] Xing X., Li D., Chen S., Wang L., Li Z., He L. (2020). Evaluation of left ventricular systolic function in patients with different types of ischemic heart disease by two-dimensional speckle tracking imaging. J. Cardiothorac. Surg..

[37] Bolger A.F., Heiberg E., Karlsson M., Wigström L., Engvall J., Sigfridsson A., Ebbers T., Kvitting J.-P.E., Carlhäll C.J., Wranne B. (2007). Transit of blood flow through the human left ventricle mapped by cardiovascular magnetic resonance. J. Cardiovasc. Magn. Reson..

[38] Zajac J., Eriksson J., Alehagen U., Ebbers T., Bolger A.F., Carlhäll C.-J. (2018). Mechanical dyssynchrony alters left ventricular flow energetics in failing hearts with LBBB: A 4D flow CMR pilot study. Int. J. Cardiovasc. Imaging.

[39] McDonagh T.A., Metra M., Adamo M., Gardner R.S., Baumbach A., Böhm M., Burri H., Butler J., Čelutkienė J., Chioncel O. (2022). 2021 ESC Guidelines for the diagnosis and treatment of acute and chronic heart failure: Developed by the Task Force for the diagnosis and treatment of acute and chronic heart failure of the European Society of Cardiology (ESC) With the special contribution of the Heart Failure Association (HFA) of the ESC. Eur. J. Heart Fail..

[40] Abraham W.T., Fisher W.G., Smith A.L., Delurgio D.B., Leon A.R., Loh E., Kocovic D.Z., Packer M., Clavell A.L., Hayes D.L. (2002). Cardiac resynchronization in chronic heart failure. N. Engl. J. Med..

[41] Lim T.T., Nickels T.B., Green S.I. (1995). Vortex Rings. Fluid Vortices.

[42] Akhmetov D.G. (2009). Introduction. Vortex Rings.

[43] Kheradvar A., Pedrizzetti G. (2012). Vortex formation in the heart. Vortex Formation in the Cardiovascular System.

[44] Mitter S.S., Shah S.J., Thomas J.D. (2017). A test in context: E/A and e/e’ to assess diastolic dysfunction and LV filling pressure. J. Am. Coll. Cardiol..

[45] Calkoen E.E., Elbaz M.S.M., Westenberg J.J.M., Kroft L.J.M., Hazekamp M.G., Roest A.A.W., van der Geest R.J. (2015). Altered left ventricular vortex ring formation by 4-dimensional flow magnetic resonance imaging after repair of atrioventricular septal defects. J. Thorac. Cardiovasc. Surg..

[46] Schäfer M., Humphries S., Stenmark K.R., Kheyfets V.O., Buckner J.K., Hunter K.S., Fenster B.E. (2018). 4D-flow cardiac magnetic resonance-derived vorticity is sensitive marker of left ventricular diastolic dysfunction in patients with mild-to-moderate chronic obstructive pulmonary disease. Eur. Heart J. Cardiovasc. Imaging.

[47] Elbaz M.S.M., van der Geest R.J., Calkoen E.E., de Roos A., Lelieveldt B.P.F., Roest A.A.W., Westenberg J.J.M. (2017). Assessment of viscous energy loss and the association with three-dimensional vortex ring formation in left ventricular inflow: In vivo evaluation using four-dimensional flow MRI. Magn. Reson. Med..

[48] Arvidsson P.M., Töger J., Carlsson M., Steding-Ehrenborg K., Pedrizzetti G., Heiberg E., Arheden H. (2017). Left and right ventricular hemodynamic forces in healthy volunteers and elite athletes assessed with 4D flow magnetic resonance imaging. Am. J. Physiol. Heart Circ. Physiol..

[49] Eriksson J., Bolger A.F., Ebbers T., Carlhäll C.-J. (2016). Assessment of left ventricular hemodynamic forces in healthy subjects and patients with dilated cardiomyopathy using 4D flow MRI. Physiol. Rep..

[50] Eriksson J., Zajac J., Alehagen U., Bolger A.F., Ebbers T., Carlhäll C.-J. (2017). Left ventricular hemodynamic forces as a marker of mechanical dyssynchrony in heart failure patients with left bundle branch block. Sci. Rep..

[51] Zajac J., Eriksson J., Dyverfeldt P., Bolger A.F., Ebbers T., Carlhäll C.-J. (2015). Turbulent kinetic energy in normal and myopathic left ventricles. J. Magn. Reson. Imaging.

[52] Kaur H., Assadi H., Alabed S., Cameron D., Vassiliou V.S., Westenberg J.J.M., van der Geest R., Zhong L., Dastidar A., Swift A.J. (2020). Left ventricular blood flow kinetic energy assessment by 4D flow cardiovascular magnetic resonance: A systematic review of the clinical relevance. J. Cardiovasc. Dev. Dis..

[53] Carlhäll C.J., Bolger A. (2010). Passing strange: Flow in the failing ventricle. Circ. Heart Fail..

[54] Crandon S., Elbaz M.S.M., Westenberg J.J.M., van der Geest R.J., Plein S., Garg P. (2017). Clinical applications of intra-cardiac four-dimensional flow cardiovascular magnetic resonance: A systematic review. Int. J. Cardiol..

[55] Lewandowski A.J., Raman B., Banerjee R., Milanesi M. (2017). Novel Insights into Complex Cardiovascular Pathologies using 4D Flow Analysis by Cardiovascular Magnetic Resonance Imaging. Curr. Pharm. Des..

[56] Zhong L., Schrauben E.M., Garcia J., Uribe S., Grieve S.M., Elbaz M.S., Barker A.J., Geiger J., Nordmeyer S., Marsden A. (2019). Intracardiac 4D flow MRI in congenital heart disease: Recommendations on behalf of the ISMRM flow & motion study group. J. Magn. Reson. Imaging.

