# Supplementary material for: Evaluation of Left Ventricular Function Using Four-Dimensional Flow Cardiovascular Magnetic Resonance: A Systematic Review
Source: J Cardiovasc Dev Dis. 2022 Sep 12;9(9):304. doi: 10.3390/jcdd9090304 (PMC9503592; doi:10.3390/jcdd9090304)
Supplement: Supplementary file 1 [file jcdd-09-00304-s001.zip › jcdd-1894902-supplementary.pdf]

Table S1. Study list, clinical applicability, population demographics, and quantitative parameters.

Study	Clinical applicability score (%)	Study population	Comparator			Control			Quantitative parameters
			Age±stdv or range (years)	Total cases	Male cases	Age±stdv or range (years)	Total cases	Male cases	
Arvidsson et al. 2017 [48]	83	2 patients with dilated cardiomyopathy vs. 14 Elite athletes vs. 25 healthy controls	Athletes: 24 (18-30) Patient 1: 58 (F) Patient 2: 71 (M)	Athletes: 14 Patients: 2	Athletes: 6 Patients: 1	28 (23-63)	25	12	Haemodynamic forces
Arvidsson et al. 2018 [16]	67	LV dyssynchrony in CRT patients vs. healthy and elite athlete controls	67	31	24	27	39	18	Haemodynamic forces
Bolger et al. 2007 [37]	67	Healthy	None	None	None	44±14	17	11	Flow components; Flow components KE
Calkoen et al. 2015 [45]	83	Patients with corrected AVSD vs. healthy control	26±12	32	9	23 (13-38)	30	14	Vortex morphology; Vortex formation time index;
Carlsson et al. 2012 [21]	67	Healthy	None	None	None	30±11	9	6	KE
Corrado et al. 2019 [13]	83	Anterior AMI patients vs healthy controls	66±12	12	11	40±16	19	10	Flow components; KE indexed to LVEDV;
Costello et al. 2018 [3]	83	Dilated cardiomyopathy vs. healthy control	54±14	16	10	43±11	16	9	Residence time distribution
Crandon et al. 2018 [23]	100	Healthy	None	None	None	45±17	53	32	KE indexed to LVEDV
Elbaz et al. 2014 [26]	100	Healthy	None	None	None	20±10	24	9	Vortex morphology
Elbaz et al. 2017 [47]	83	Patients with corrected AVSD vs. healthy control	26±12	32	9	23 (13-38)	30	14	EL indexed to SV
Eriksson et al.	83	Dilated cardiomyopathy vs. healthy control	49±14	10	4	48±15	10	6	Haemodynamic forces

Study	Clinical applicability score (%)	Study population	Comparator			Control			Quantitative parameters
			Age±stdv or range (years)	Total cases	Male cases	Age±stdv or range (years)	Total cases	Male cases	
2016 [49]									
Eriksson et al. 2017 [50]	83	Patients with ischaemic or dilated cardiomyopathy with vs. without LBBB	63±11	9	8	59±14	9	8	Haemodynamic forces
Garg et al. 2018 [24]	100	Post acute MI or chronic IHD vs. healthy controls	57±11	48	39	52.3±1.2	20	12	KE indexed to LVEDV
Garg et al. 2019 [17]	100	Post acute MI or chronic IHD with or without LV thrombus vs. healthy controls	MI with thrombus: 61±13 MI without thrombus: 60±9	MI with thrombus: 32 MI without thrombus: 36	MI with thrombus: 29 MI without thrombus: 28	Age-matched controls: 57±7 Younger controls: 30±10	40	25	KE indexed to LVEDV
Kamphuis et al. 2018 [19]	83	Healthy	None	None	None	27±3	12	6	KE; EL raw value and indexed to SV; Vorticity
Kanski et al. 2015 [30]	83	Heart failure vs. healthy controls	67±8	29	24	27±3	12	8	KE raw value and indexed to SV and LVEDV; Vortex KE
Miyajima et al. 2021 [14]	100	Patients with LBBB vs without LBBB	71 (66-76)	16	7	65 (54-76)	16	11	EL; Vortex morphology
Nakaji et al. 2021 [22]	83	Healthy	None	None	None	29.9±5	19	11	KE indexed to BSA; EL indexed to BSA
Pewowaruk et al. 2021 [32]	67	Healthy	None	None	None	NA	20	10	KE raw value and indexed to SV and mask volume, dimensionless KE; EL raw value and indexed to SV and mask volume, dimensionless EL;

Study	Clinical applicability score (%)	Study population	Comparator			Control			Quantitative parameters
			Age±stdv or range (years)	Total cases	Male cases	Age±stdv or range (years)	Total cases	Male cases	
									Vorticity
Rutkowski et al. 2020 [31]	67	Healthy males vs. healthy females	26±2.8	20	20	27±2.9	19	19	KE raw value and indexed to SV Vorticity
Schäfer et al. 2016 [27]	100	Pulmonary hypertension vs control	64 (59-70)	13	3	58.5 (53.7-65.5)	10	3	Vorticity
Schäfer et al. 2018 [46]	83	COPD with presumed LV diastolic dysfunction vs healthy control	65±2	16	10	57±9	10	7	Vorticity
Steding-Ehrenborg et al. 2016 [28]	83	Athletes vs sedentary healthy controls	Male: 23±3 Female: 25±5	14	6	Male: 26±2 Female: 33±10	14	8	KE
Stoll et al. 2018 [20]	83	Healthy	None	None	None	54±14	45	27	Flow components; Flow components KE
Sundin et al. 2020 [25]	83	Healthy pre- vs. post-Dobutamine	None	None	None	33±13	12	4	Flow components volume indexed to LVEDV; Flow components KE indexed to LVEDV
Suwa et al. 2016 [18]	100	Impaired LV function vs. preserved LV function patients	64±10.1	14	11	61.2±14.3	21	10	Vortex morphology
Svalbring et al. 2016 [8]	83	Chronic ischaemic heart disease with no to mild LV systolic dysfunction and remodeling vs. healthy control	68±5	26	16	62±11	10	3	Flow components, indexed to LVEDV; Flow components KE, indexed to LVEDV
Wong et al. 2016 [15]	67	LV systolic dysfunction vs healthy adult and children control	51±15	10	5	29±13	35	19	KE raw value and indexed to LV mass

Study	Clinical applicability score (%)	Study population	Comparator			Control			Quantitative parameters
			Age±stdv or range (years)	Total cases	Male cases	Age±stdv or range (years)	Total cases	Male cases	
Zajac et al. 2015 [51]	83	Dilated cardiomyopathy vs. healthy control	51±13	9	4	43±18	11	6	TKE
Zajac et al. 2018 [38]	83	Heart failure patients with vs. without LBBB	61±14	11	9	58±16	11	9	Flow components; Flow components KE

AMI: acute myocardial infarction; AVSD: atrioventricular septal defect; BSA: body surface area; COPD: chronic obstructive pulmonary disease; CRT: cardiac resynchronisation therapy; EL: energy loss; IHD: ischaemic heart disease; KE: kinetic energy; LBBB: left bundle branch block; LVEDV: left ventricle end-diastolic volume; SV: stroke volume; TKE: turbulent kinetic energy.

Table S2. 4D-flow MRI sequence parameters.

Study	Field (Tesla)	Scanner type	Acceleration		Sampling resolution (mm)		Acquired temporal resolution (ms)	TE/TR (ms)	Flip angle (°)	No. of reconstructed phases (n)	VENC (cm/s)	Scan time (min)	Respiratory gating	Cardiac gating
			Type	Factor	In-Plane	Slice								
Arvidsson et al. 2017 [48]	1.5/3	Philips Achieva	SENSE	2	3	3	50	3.7/6.3	8	NA	100	NA	Yes (navigator)	Retrospective
Arvidsson et al. 2018 [16]	1.5/3	Philips Achieva	PI	2	3	3	50	3.1-3.7/5.1-6.3	8	40	NA	28+/-7	Yes (navigator)	Retrospective
Bolger et al. 2007 [37]	1.5	GE Signa	NA	NA	1-4	4-8	72	6/18	20	32	60	30	NA	NA
Calkoen et al. 2015 [45]	3	Philips Ingenia	SENSE	2	2.3	3.0-4.2	31	3.2/7.7	10	30	150	8-10	No	Retrospective
Carlsson et al. 2012 [21]	3	Philips Intera	SENSE	2	3	3	NA	3.7/6.3	8	NA	NA	NA	Yes (navigator)	Retrospective
Corrado et al. 2019 [13]	1.5/3	GE MR450w, HDxt, MR750 or MR750w	PC-VIPR	NA	1.25	1.25	NA	2.0-2.5/5.8-8.4	8-12	20	100-150	9-14	Yes	Retrospective
Costello et al. 2018 [3]	3	Siemens Prisma	NA	NA	3	3	NA	NA/42.5	NA	20	200	6-12	Yes	Yes (not specified)
Crandon et al. 2018 [23]	1.5	Philips Ingenia	EPI	5	3	3	40	3.5/10	10	30	150	NA	No	Retrospective
Elbaz et al. 2014 [26]	3	Philips Ingenia	EPI	5	2.3	3.0-4.2	NA	3.0/9.9	10	30	150	NA	No	Retrospective

Study	Field (Tesla)	Scanner type	Acceleration		Sampling resolution (mm)		Acquired temporal resolution (ms)	TE/TR (ms)	Flip angle (◦)	No. of reconstructed phases (n)	VENC (cm/s)	Scan time (min)	Respiratory gating	Cardiac gating
			Type	Factor	In-Plane	Slice								
Elbaz et al. 2017 [47]	3	Philips Ingenia	EPI	5	2.3	3.0-4.2	31	3.2/7.7	10	30	150	NA	No	Retrospective
Eriksson et al. 2016 [49]	1.5	Philips Achieva	SENSE	2	3	3	50.4	3.7/6.3	8	40	100	16-57	Yes (navigator)	Retrospective
Eriksson et al. 2017 [50]	3	Philips Ingenia	SENSE	3	2.8	2.8	52.8	2.6/4.4	5 or 10	40	120	16+/-6.9	Yes (navigator)	Retrospective
Garg et al. 2018 [24]	1.5	Philips Ingenia	EPI	5	3	3	40	3.5/10	10	30	150	NA	No	Retrospective
Garg et al. 2019 [17]	1.5	Philips Ingenia	EPI	5	3	3	40	3.5/10	10	30	150	NA	No	Retrospective
Kamphuis et al. 2018 [19]	3	Philips Ingenia	SENSE	2	3	3	40	3.7/10	10	30	150	9	No	Retrospective
Kanski et al. 2015 [30]	1.5/3	Philips Achieva	SENSE	2	3.0	3.0	50-55	3.7/6.2	8	40	100	NA	No	Retrospective
Miyajima et al. 2021 [14]	1.5/3	Philips Provida or Philips Ingenia	SENSE	3.5	1.29-1.97	4	48.1-90.2	1.87-1.96/3.4-3.6	10	20	250	NA	Yes (navigator)	Retrospective
Nakaji et al. 2021 [22]	3	Siemens Skyra	GRA-PPA	2	1.8	4	NA	2.86/67.8	8	13-20	150	12-25	No	Prospective
Pewowaruk et al. 2021 [32]	3	GE MR750 or Signa Premier	PC-VIPR	NA	1.25	1.25	NA	NA/6.2	NA	14	150	11	Yes (Bellow)	Retrospective

Study	Field (Tesla)	Scanner type	Acceleration		Sampling resolution (mm)		Acquired temporal resolution (ms)	TE/TR (ms)	Flip angle (◦)	No. of reconstructed phases (n)	VENC (cm/s)	Scan time (min)	Respiratory gating	Cardiac gating
			Type	Factor	In-Plane	Slice								
Rutkowski et al. 2020 [31]	3	GE MR750 and Signa Premier	PC-VIPR	NA	1.25	1.25	NA	NA/6.2	NA	14	160	11	Yes (Bellow)	Retrospective
Schäfer et al. 2016 [27]	1.5	Siemens Avanto	NA	NA	2.3-2.7	2.3-2.7	50	2.85/48.56	15	10-30	100	NA	Yes (Bellow)	Prospective
Schäfer et al. 2018 [46]	1.5	Siemens Avanto	NA	NA	2.4-2.6	2.4-3.0	NA	2.85/48.56	14-15	NA	100-150	NA	Yes (Bellow)	Prospective
Steding-Ehrenborg et al. 2016 [28]	1.5/3	Philips Achieva	SENSE	2	3	3	50	3.7/6.3	8	NA	NA	NA	Yes (navigator)	Retrospective
Stoll et al. 2018 [20]	3	Siemens Trio	NA	NA	3	3	52	2.75/4.3	7	NA	100	15-20	Yes (navigator)	Retrospective
Sundin et al. 2020 [25]	3	Philips Ingenia	SENSE	1.6-3	2.8	2.8	40	2.6/5.2	5	NA	140	7-8	Yes (navigator)	Retrospective
Suwa et al. 2016 [18]	3	GE MR750	ARC	2	2	2	NA	2.0/4.5-5.0	15	20	200	10	Yes	Retrospective
Svalbring et al. 2016 [8]	3	Philips Ingenia	SENSE	3	2.8	2.8	52.8	2.6/4.3	10	NA	120	7-9	Yes (navigator)	Retrospective
Wong et al. 2016 [15]	1.5	Philips Achieva	k-t PCA	8	2.0-3.0	2.0-3.0	<35	2.4/3.8	5	24-32	NA	5-7	Yes	Prospective
Zajac et al. 2015 [51]	1.5	Philips Achieva	SENSE	2	3	3	NA	3.7/6.3	8	NA	100	10-15	Yes (navigator)	Retrospective

Study	Field (Tesla)	Scanner type	Acceleration		Sampling resolution (mm)		Acquired temporal resolution (ms)	TE/TR (ms)	Flip angle (◦)	No. of reconstructed phases (n)	VENC (cm/s)	Scan time (min)	Respiratory gating	Cardiac gating
			Type	Factor	In-Plane	Slice								
Zajac et al. 2018 [38]	3	Philips Ingenia	SENSE	3	2.8	2.8	52.8	2.6/4.4	5 or 10	40	120	17+/-7	Yes (navigator)	Retrospective

TE: echo time; TR: repetition time; EPI: echo planar imaging; PI: parallel imaging; VIPR: isotropic-voxel radial projection imaging; SENSE: sensitivity encoding; GRAPPA: generalised autocalibrating partial parallel acquisition; ARC: Autocalibrating Reconstruction for Cartesian imaging; VENC: velocity encoding; Scanner manufacturers: Achieva, Provida, Intera, Ingenia: Philips Healthcare, Best, Netherlands; Avanto, Prisma, Skyra, Trio: Siemens Healthineers, Erlangen, Germany; Signa, MR450w, MR750, MR 750w, HDxt: General Electric Healthcare, Milwaukee, Wisconsin, USA.
